# Artificial intelligence-enabled electrocardiogram to distinguish cavotricuspid isthmus dependence from other atrial tachycardia mechanisms^[Author-notes ztac042-FM1]^

**DOI:** 10.1093/ehjdh/ztac042

**Published:** 2022-08-17

**Authors:** Arunashis Sau, Safi Ibrahim, Amar Ahmed, Balvinder Handa, Daniel B Kramer, Jonathan W Waks, Ahran D Arnold, James P Howard, Norman Qureshi, Michael Koa-Wing, Daniel Keene, Louisa Malcolme-Lawes, David C Lefroy, Nicholas W F Linton, Phang Boon Lim, Amanda Varnava, Zachary I Whinnett, Prapa Kanagaratnam, Danilo Mandic, Nicholas S Peters, Fu Siong Ng

**Affiliations:** National Heart and Lung Institute, Imperial College London, Du Cane Road, London W12 0NN, UK; Department of Cardiology, Hammersmith Hospital, Imperial College Healthcare NHS Trust, Du Cane Road, London W12 0NN, UK; National Heart and Lung Institute, Imperial College London, Du Cane Road, London W12 0NN, UK; National Heart and Lung Institute, Imperial College London, Du Cane Road, London W12 0NN, UK; National Heart and Lung Institute, Imperial College London, Du Cane Road, London W12 0NN, UK; Department of Cardiology, Hammersmith Hospital, Imperial College Healthcare NHS Trust, Du Cane Road, London W12 0NN, UK; National Heart and Lung Institute, Imperial College London, Du Cane Road, London W12 0NN, UK; Richard A. and Susan F. Smith Center for Outcomes Research in Cardiology, Beth Israel Deaconess Medical Center, Harvard Medical School, 330 Brookline Avenue, Boston, MA 02215, USA; Harvard-Thorndike Electrophysiology Institute, Beth Israel Deaconess Medical Center, Harvard Medical School, 330 Brookline Avenue, Boston, MA 02215, USA; National Heart and Lung Institute, Imperial College London, Du Cane Road, London W12 0NN, UK; Department of Cardiology, Hammersmith Hospital, Imperial College Healthcare NHS Trust, Du Cane Road, London W12 0NN, UK; National Heart and Lung Institute, Imperial College London, Du Cane Road, London W12 0NN, UK; Department of Cardiology, Hammersmith Hospital, Imperial College Healthcare NHS Trust, Du Cane Road, London W12 0NN, UK; National Heart and Lung Institute, Imperial College London, Du Cane Road, London W12 0NN, UK; Department of Cardiology, Hammersmith Hospital, Imperial College Healthcare NHS Trust, Du Cane Road, London W12 0NN, UK; Department of Cardiology, Hammersmith Hospital, Imperial College Healthcare NHS Trust, Du Cane Road, London W12 0NN, UK; National Heart and Lung Institute, Imperial College London, Du Cane Road, London W12 0NN, UK; Department of Cardiology, Hammersmith Hospital, Imperial College Healthcare NHS Trust, Du Cane Road, London W12 0NN, UK; Department of Cardiology, Hammersmith Hospital, Imperial College Healthcare NHS Trust, Du Cane Road, London W12 0NN, UK; Department of Cardiology, Hammersmith Hospital, Imperial College Healthcare NHS Trust, Du Cane Road, London W12 0NN, UK; National Heart and Lung Institute, Imperial College London, Du Cane Road, London W12 0NN, UK; Department of Cardiology, Hammersmith Hospital, Imperial College Healthcare NHS Trust, Du Cane Road, London W12 0NN, UK; National Heart and Lung Institute, Imperial College London, Du Cane Road, London W12 0NN, UK; Department of Cardiology, Hammersmith Hospital, Imperial College Healthcare NHS Trust, Du Cane Road, London W12 0NN, UK; Department of Cardiology, Hammersmith Hospital, Imperial College Healthcare NHS Trust, Du Cane Road, London W12 0NN, UK; National Heart and Lung Institute, Imperial College London, Du Cane Road, London W12 0NN, UK; Department of Cardiology, Hammersmith Hospital, Imperial College Healthcare NHS Trust, Du Cane Road, London W12 0NN, UK; National Heart and Lung Institute, Imperial College London, Du Cane Road, London W12 0NN, UK; Department of Cardiology, Hammersmith Hospital, Imperial College Healthcare NHS Trust, Du Cane Road, London W12 0NN, UK; Department of Electrical and Electronic Engineering, Imperial College London, South Kensington Campus, Exhibition Road, London SW7 2AZ, UK; National Heart and Lung Institute, Imperial College London, Du Cane Road, London W12 0NN, UK; Department of Cardiology, Hammersmith Hospital, Imperial College Healthcare NHS Trust, Du Cane Road, London W12 0NN, UK; National Heart and Lung Institute, Imperial College London, Du Cane Road, London W12 0NN, UK; Department of Cardiology, Hammersmith Hospital, Imperial College Healthcare NHS Trust, Du Cane Road, London W12 0NN, UK

**Keywords:** Atrial tachycardia, Atrial flutter, Electrocardiogram, Artificial intelligence, Machine learning

## Abstract

**Aims:**

Accurately determining atrial arrhythmia mechanisms from a 12-lead electrocardiogram (ECG) can be challenging. Given the high success rate of cavotricuspid isthmus (CTI) ablation, identification of CTI-dependent typical atrial flutter (AFL) is important for treatment decisions and procedure planning. We sought to train a convolutional neural network (CNN) to classify CTI-dependent AFL vs. non-CTI dependent atrial tachycardia (AT), using data from the invasive electrophysiology (EP) study as the gold standard.

**Methods and results:**

We trained a CNN on data from 231 patients undergoing EP studies for atrial tachyarrhythmia. A total of 13 500 five-second 12-lead ECG segments were used for training. Each case was labelled CTI-dependent AFL or non-CTI-dependent AT based on the findings of the EP study. The model performance was evaluated against a test set of 57 patients. A survey of electrophysiologists in Europe was undertaken on the same 57 ECGs. The model had an accuracy of 86% (95% CI 0.77–0.95) compared to median expert electrophysiologist accuracy of 79% (range 70–84%). In the two thirds of test set cases (38/57) where both the model and electrophysiologist consensus were in agreement, the prediction accuracy was 100%. Saliency mapping demonstrated atrial activation was the most important segment of the ECG for determining model output.

**Conclusion:**

We describe the first CNN trained to differentiate CTI-dependent AFL from other AT using the ECG. Our model matched and complemented expert electrophysiologist performance. Automated artificial intelligence-enhanced ECG analysis could help guide treatment decisions and plan ablation procedures for patients with organized atrial arrhythmias.

## Graphical Abstract

**Graphical Abstract:**
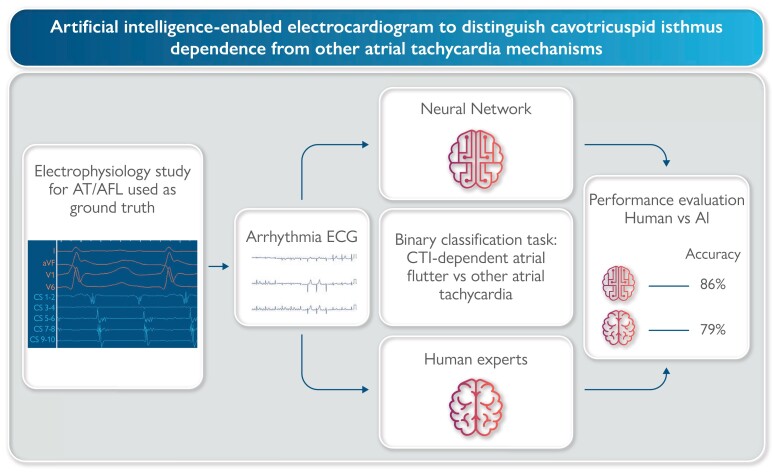

## Introduction

There are multiple forms of organized atrial arrhythmias, which may have focal, macro-reentrant or micro-reentrant mechanisms.^1^ They are defined by the location of the focal source or circuit, of which cavotricuspid isthmus (CTI)-dependent atrial ‘typical’ flutter (hereby referred to as AFL) is the most common.^2^ Catheter ablation is a highly effective therapy for CTI-dependent AFL and should be considered a first-line approach.^3^ There has been a sharp increase in atrial fibrillation (AF) ablation over the last decade,^4^ and atrial arrhythmia recurrence post-AF ablation often occurs in an organized form: either as a macroreentrant atrial tachycardia (MRAT), particularly roof- and mitral- isthmus dependent MRATs, or as micro-reentrant tachycardias around localized scar.^5^ Catheter ablation of these atrial tachycardias (ATs) is also effective, but success rates are lower given the diverse range of potential mechanisms, and the risks higher given the need for left atrial access.^5^

CTI-dependent AFL can be characterized by the sawtooth appearance in the inferior leads of the 12-lead electrocardiogram (ECG).^6^ However, it is often difficult to accurately determine CTI-dependence on a surface ECG alone.^7^ Pre-procedural knowledge of CTI dependence vs. non-CTI dependent mechanism has important implications for treatment decisions and planning an ablation procedure. Given the greater than 90% single procedure success rate following CTI ablation,^8^ physicians and patients are likely to have a lower threshold to pursuing an invasive strategy. Conversely, the threshold for an invasive strategy for non-CTI dependent AT (subsequently referred to as AT) is higher. This is due to the lower success rates and potential need for transeptal puncture.^9,10^ Knowledge of the tachycardia mechanism can also help planning the procedure. Non-CTI-dependent ATs are often left-sided,^9^ requiring a transseptal puncture, for which a general anaesthetic and transoesophageal (TOE) guidance may be needed. Lastly, given the increased risks of a transeptal puncture and left sided AT ablation, knowledge of arrhythmia mechanism prior to the procedure can help when counselling the patient regarding the risks of the procedure.

Machine learning, with convolutional neural networks (CNNs) in particular, has been used to classify arrhythmias, using the 12-lead ECG, with great accuracy.^11–13^ However, most studies use human interpretation of the ECG as the ground truth to label the arrhythmia ECGs. Therefore, these neural networks can only ever be as good as expert human interpretation. Machine learning models have also been trained on ECGs using results from another test as the ground truth,^14–17^ in order to allow the models to surpass human performance. We hypothesized a CNN could be trained to match or even exceed expert human performance in classifying CTI-dependent AFL vs. non-CTI-dependent AT, when using findings from the invasive electrophysiology (EP) study as the gold standard ground truth.

## Methods

### Ethics

This retrospective study was approved by the regional ethics board (IRAS ID: 293374).

### Patient selection

**Figure 1:**
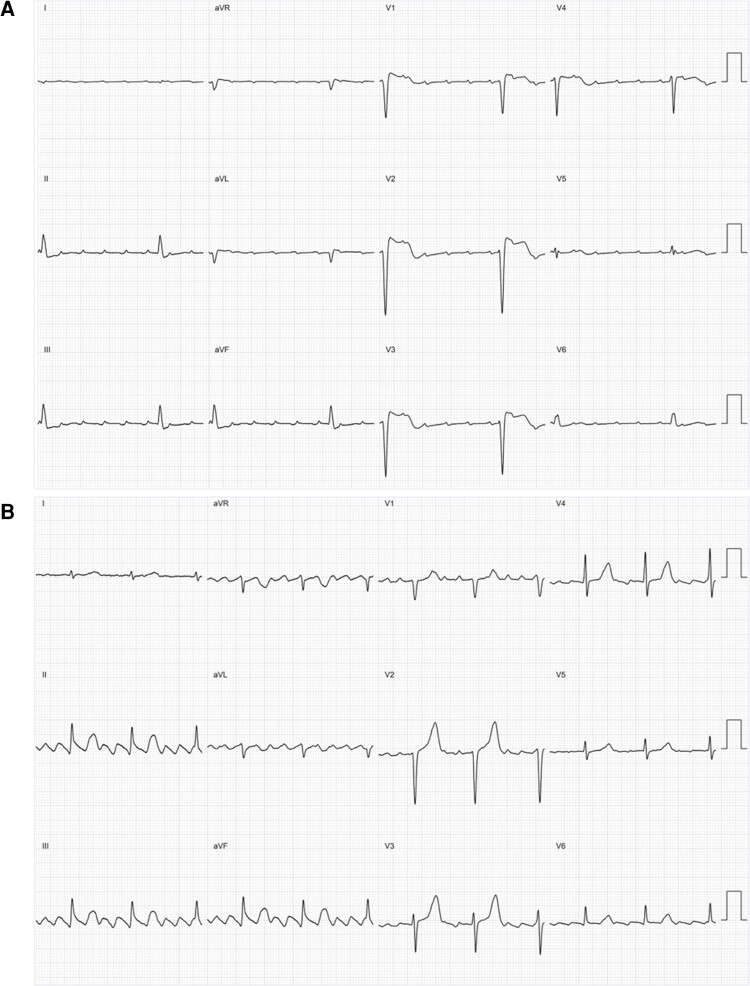
Electrocardiogram class examples. Example 12-lead electrocardiograms of the classes used to train the neural network. An electrocardiogram showing (*A*) non-cavotricuspid isthmus dependent atrial tachycardia (*B*) cavotricuspid isthmus-dependent atrial flutter.

Patients were selected from a clinical database of all invasive EP procedures at Hammersmith Hospital, Imperial College Healthcare NHS Trust, United Kingdom. A search was performed for any AT, atrial flutter/CTI ablations for the years 2012–21. The patients all had procedures using an EP recording system, LABSYSTEM™ PRO (Boston Scientific). The ECG and the intracardiac electrogram recordings for each case (pre-ablation) were visually examined for lengths that contained sustained tachycardias without diagnostic pacing manoeuvres or excessive noise. The intracardiac electrograms were inspected to ensure the selected segments contained AT/AFL and not another tachycardia (e.g. sinus tachycardia or atrial fibrillation). Using the EP recording system, digital 12-lead ECG recordings were exported (*Figure 1*).

### Data labelling

For each case, the procedure report was reviewed to determine the diagnosis. Diagnoses were made by the clinical team carrying out the ablation procedure and generally were based on 3D mapping (where used) and/or entrainment and/or termination with ablation. The final diagnoses included all types of organized left and right atrial tachyarrhythmia, including clockwise and counter-clockwise CTI-dependent AFL, non-CTI-dependent macro re-entrant, focal, and micro-reentrant tachycardias. Atrial fibrillation was not included. 3D mapping was used in some but not all cases. In particular, many CTI ablation cases did not use 3D mapping, and in those cases, the diagnosis was confirmed by arrhythmia termination during CTI ablation, by entrainment, or both. Cases with little or no sustained arrhythmia or without a clear mechanism diagnosed were excluded. For this proof-of-concept study, a small number of patients diagnosed with CTI-dependent AFL following cardiac surgery, other than isolated coronary artery bypass grafting (CABG), were excluded.

### ECG extraction

Digital 12-lead surface ECGs were extracted at a sampling rate of 1000 Hz. For patients with multiple procedures, within 2012–21, only one procedure was included. Patients were randomly assigned to training and testing datasets. In the training set, each patient had up to 5 min of data exported in up to one-minute segments. Each one-minute segment was further split into 5 s segments. Segmentation into 5 s segments was performed in an automated fashion, with no visual inspection steps. Given the likely significant similarities between consecutive 5 s recordings of an organized atrial rhythm, and for fair comparison with human performance, the test set used only one 5 second segment for each patient. Importantly, the no data from the test dataset was used in training dataset and the patients in the test set were distinct to those in the training set. A data flow chart is shown in Supplementary material online, *Figure S1*.

### Data pre-processing

Multichannel exports from the EP recording system were converted into time series data.^18^ Each ECG recording was downsampled to 400 Hz. This allowed a reduction in the dataset size, thereby reducing model training time while maintaining accuracy.

### CNN structure, training and testing

CNNs are a type of deep learning based on biological neural networks.^19^ The convolutional layers detect features on the input data that can be used in subsequent layers of the neural network to predict a classification for the input data.

In this work, a CNN was trained on 12-lead ECGs for arrhythmia classification. Several different 1D CNN architectures, based on previous work, were implemented and their performance compared using a single subset of the training set as a validation set^11–13,16,20^ (see Supplementary material online, *Methods* and *Table S1*). This included ResNet based architectures such as ResNet34.^21^ For hyperparameter tuning 20% of the training dataset was used as a validation set. The Keras Tuner package (version 1.0.2) was used for hyperparameter tuning within each architecture against the validation dataset.^22^ The hyperparameters tuned were the learning rate, batch size, dropout, and filter size. The test dataset was not used during model selection or hyperparameter tuning. A model training flow chart is shown in Supplementary material online, *Figure S2*.

**Figure 2:**
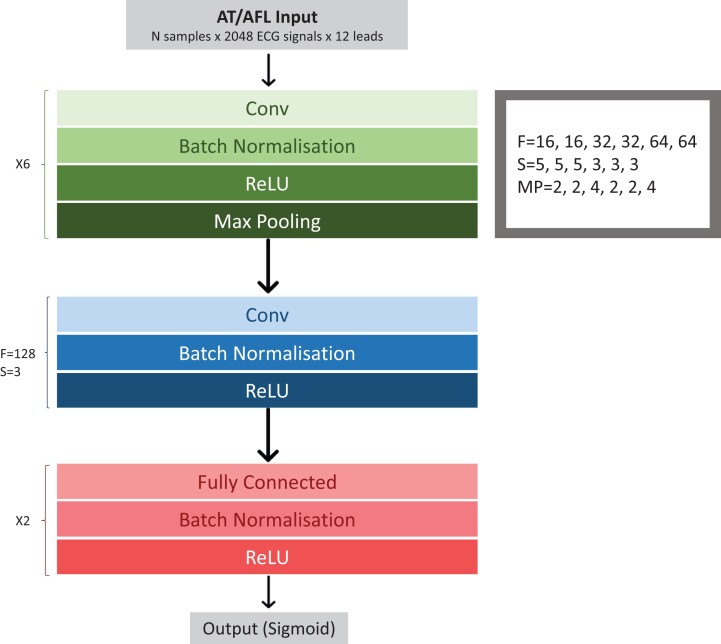
Neural network architecture. Selected model architecture is shown. Our final network was a modified version of one used by an Attia *et al*.^16^ It is comprised of a total of seven convolutional layers with two fully connected layers. AT, non-cavotricuspid isthmus dependent atrial tachycardia; AFL, CTI dependent atrial flutter; Conv, convolutional layer; ReLU, rectified linear unit; F, number of filters; S, kernel size; MP, maxpool factor.

Our final network was selected based on performance in the validation set, this was a modified version of that used by Attia *et al*.^16^ and is comprised of a total of thirty-three layers (*Figure 2*). The remaining tested networks, including those using residual blocks were inferior to this network when evaluated using the validation dataset. The complete architecture of the neural network is detailed in the figure.

The model input was 12-lead ECGs of 5-s duration. The ECG data was zero padded to 2048 × 12. The learning rate was 0.01. The binary cross-entropy was minimized using the Adam optimizer. The neural network was trained for a maximum of 40 epochs with early termination set to when there was no further improvement in validation set loss for seven consecutive epochs. The training batch size was 16. During hyperparameter tuning, the optimal value for dropout was found to be 0, therefore the dropout layer was removed from the final model architecture. The filter numbers and sizes are detailed in *Figure 2*. To create the final model the whole training dataset (including the 20% validation set utilized for hyperparameter tuning) was split into 10 folds. Each patient’s data were assigned to only one fold to avoid training and validation against the same patient’s data. In order to utilize the whole training dataset for model training, 10-fold cross-validation was performed. In this process a model was trained on 9 folds of data and validated on the 10th fold. This is repeated 10 times, so that each fold is used as a validation fold once, to create 10 models. The 10 models were then averaged to create an ensemble model for use on the unseen test set.

In order to explore how the neural network arrived at each classification, we used the method of saliency mapping. We used the vanilla gradient algorithm in tf-keras-vis (version 0.5.5) package in Python (version 3.9),^23^ where each data point of the ECG is mathematically tested to quantitatively determine how much it contributes to the output produced. A map can then be created where the highlighted sections are the most significant, and salient, areas. This is important as it can allow us to understand the processes by which the network chooses the class in the testing phase to ensure unexpected factors are not influencing the network.^24^

Further information concerning the ECG pre-processing and architecture selection process is detailed in the Supplementary material online, *Methods*.

### Statistical analysis

The performance of the neural network was evaluated using the overall accuracy, F1 score, and area under curve (AUC) metrics. The F1 score is the harmonic mean of the precision (positive predictive value) and recall (sensitivity) and tests the accuracy of the network. The AUC measures the area under a receiver operating characteristics (ROC) curve which plots the true positive rate (sensitivity) against the false positive rate (1—specificity). Binomial proportion confidence intervals are reported. Statistical analysis was carried out in Python (version 3.9).

### Clinician survey

Each case in the test set had a 12-lead ECG produced in the traditional format using LABSYSTEM™ PRO (Boston Scientific). These ECGs were uploaded to an online survey platform where respondents had to choose a binary option of CTI-dependent AFL or non-CTI-dependent AT. It was distributed to Consultant/Attending EP specialists and other cardiologists in Europe (all of whom were external to the EP department of Hammersmith Hospital) to compare the performance of the neural network to humans (see Supplementary material online, *Figure S3*).

There were a total of twelve respondents. These respondents included nine electrophysiologists, two cardiologists (non-electrophysiologists) and a cardiology fellow. The McNemar test was used to compare model performance with human consensus performance.

## Results

**Figure 3:**
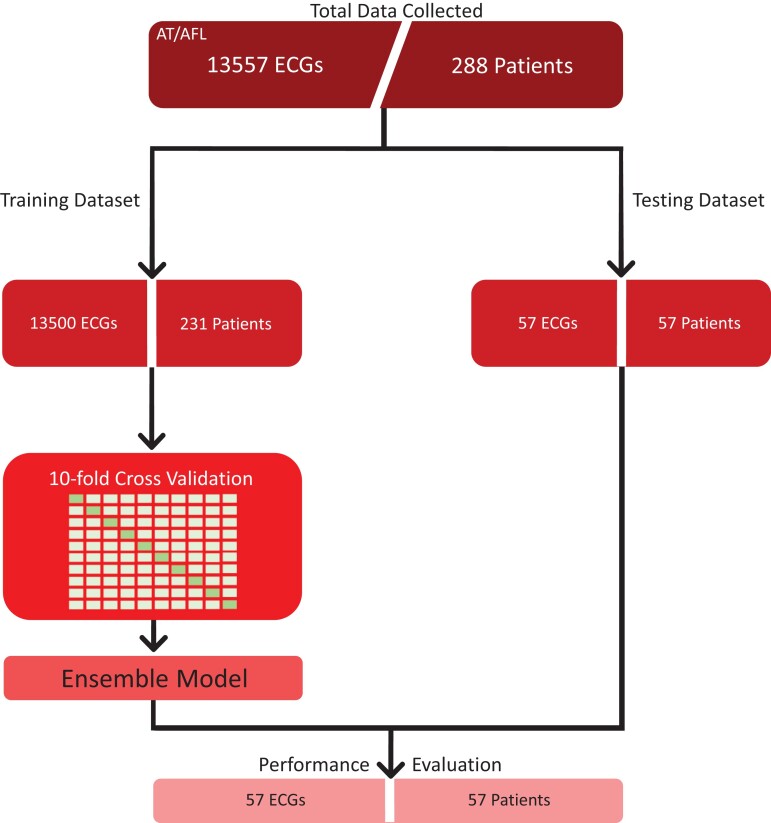
Study flowchart. Data from 288 patients were collected, 141 non-cavotricuspid isthmus-dependent atrial tachycardia cases and 147 cavotricuspid isthmus-dependent atrial flutter cases. Twelve-lead electrocardiograms were segmented into 5 second recordings. For the training dataset, multiple recordings were used per patient, and the test set used only one recording per patients. An ensemble model was created from the 10 models through cross validation. Model performance was evaluated on the unseen test set.

There were 288 patients’ ECGs analysed, 73% were male, and the average age was 71 ± 11.9 years. Patients were randomly assigned to the training and test datasets (231 in the training dataset and 57 in the testing dataset; *Figure 3*). There were a total of 141 non-CTI-dependent AT cases (subsequently referred to as AT) and 147 CTI-dependent AFL cases (subsequently referred to as AFL).

Of the 147 total AFL cases, 11 (7%) had had previous atrial ablation, of which 7 (5%) were previous CTI line ablations, 3 (2%) had had previous CABG.

Of the 141 total AT cases, 94 (67%) had had at least one previous transcatheter ablation procedures, of which 77 (55%) had had previous left atrial ablation. Nineteen (13%) of AT cases had had previous cardiac surgery, 3 (2%) were isolated CABG procedures, and 2 (1%) included the Maze procedure. The distribution of previous ablations in the training and test dataset are shown in Supplementary material online, *Table S2*.

In the test dataset, there were 29 AFL cases and 28 AT. The mean ventricular rate was 95.7 ± 30.1 bpm, there was a trend toward the AT group having a higher ventricular rate; however, this was not statistically significant (103.0 ± 31.7 bpm vs. 88.7 ± 27.2 bpm, *P* = 0.07).

### Neural network performance

**Figure 4:**
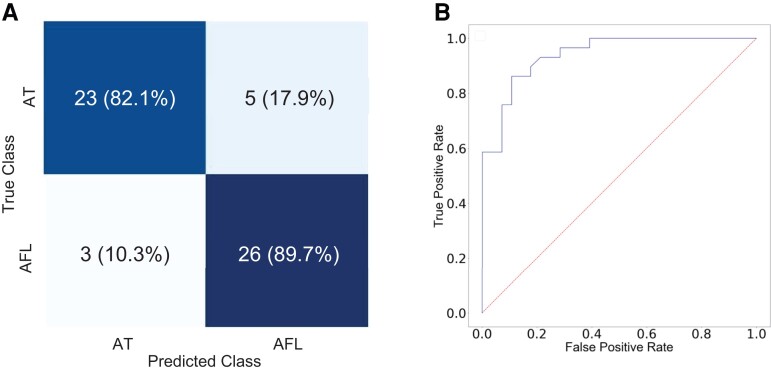
Model performance. (*A*) Confusion matrix demonstrating the accuracy of the model in classifying between the two groups. Cavotricuspid isthmus-dependent atrial flutter and non-cavotricuspid isthmus-dependent atrial tachycardia cases were correctly classified at 89.7% and 82.1%, respectively (*B*) Receiver operating characteristic curves for model performance; diagnosis of cavotricuspid isthmus-dependent atrial flutter was considered a positive case. The area under the receiver operating characteristic was 0.94.

For the testing dataset, the accuracy was 86% (95% CI 0.77–0.95). The F1 score was 0.87. *Figure 4A* displays the confusion matrix. The AT/AFL network correctly identified AT 82% and AFL 90% of the time. *Figure 4B* shows the ROC curve, the AUC was 0.94 for distinguishing the classes.

### Saliency mapping

**Figure 5:**
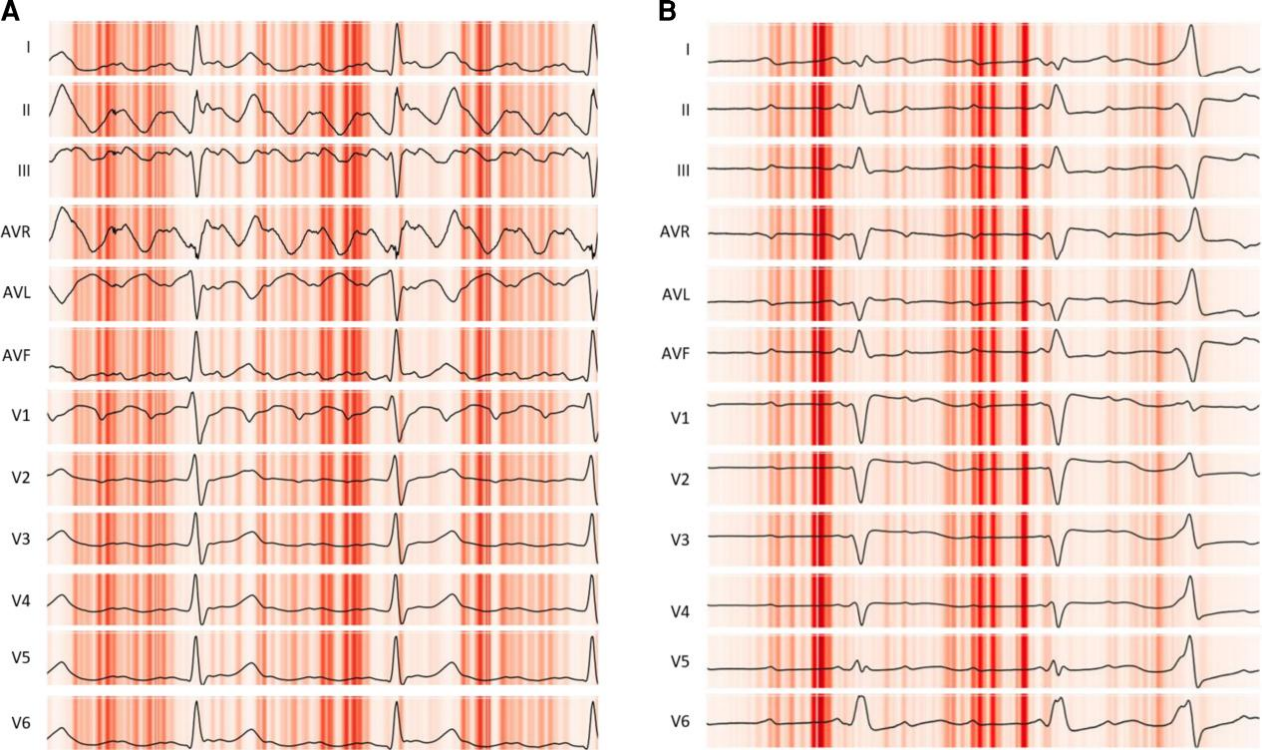
Saliency maps of each electrocardiogram class. Saliency maps showing the most prominent sections of electrocardiogram recordings in influencing the output of the neural network for the electrocardiogram classes of (*A*) cavotricuspid isthmus-dependent atrial flutter, (*B*) Non-cavotricuspid isthmus-dependent atrial tachycardia. Darker red indicates higher saliency. The atrial activity in each case appears to be salient; this is consistent with both human interpretation and the underlying mechanism of the tachycardias.

A saliency map can be used to help understand why a CNN predicted a particular outcome. This is achieved by mapping the outcome back to key areas of the input that most influenced the network in producing the classification result. *Figure 5* presents the saliency mappings of an example 12-lead ECG for each class or AFL and AT. The network used the expected sections of the ECGs for diagnoses; these were the *P*-wave segments and, in particular, not the QRS complex nor T wave. Supplementary material online, *Figures S4 and S5* give examples of saliency maps where network classification was incorrect. Notably in Supplementary material online, *Figure S4B* the network appears to place significant importance on the QRS complex, which may explain the incorrect classification in this case. For the remaining examples however, the network correctly does not focus on the QRS complex or T waves.

### Human performance comparison

When including all 12 respondents, the median accuracy was 78% with a minimum of 70% and a maximum of 86% (mean 78%, 95% CI 0.75–0.81). Specifically for the electrophysiologists, the median accuracy was 79%, minimum 70% and maximum 84% (mean 78%, 95% CI 0.75–0.81). For the non-electrophysiologists the median accuracy was 74%, minimum 72% and maximum 86% (mean 77%, 95% CI 0.58–0.96). Subsequent analyses included only electrophysiologists. There was no statistically significant difference between model performance and electrophysiologist consensus performance (electrophysiologist consensus accuracy 81%, F1 score 0.83, *P* = 0.65). Electrophysiologists were more likely to incorrectly diagnose AFL as AT (on average incorrect diagnoses: 10 AFL, 1 AT). In comparison, the neural network most often incorrectly diagnosed AT as AFL (on average incorrect diagnoses: 5 AT, 3 AFL). A confusion matrix of consensus electrophysiologist performance is shown in Supplementary material online, *Figure S6*. A contingency table comparing electrophysiologist and model performance is shown in Supplementary material online, *Table S3*. There were no cases where both the electrophysiologist consensus and model prediction were incorrect. Example cases illustrating human expert and model performance are shown in Supplementary material online, *Figures S7–S9*. In two thirds of cases (38/57, 66.67%) both the model and electrophysiologist consensus were in agreement. Human and model accuracy for these cases was 100%. For the cases where there was disagreement, electrophysiologist consensus had an accuracy of 42% (8/19 correct), while the model had 58% accuracy (11/19 correct).

## Discussion

In this study, we describe the training and testing of a CNN to classify CTI-dependent AFL vs. non-CTI-dependent AT, based on the ground truth labels derived from the gold standard of an invasive EP study. Despite a relatively small number of patients in the training dataset, our neural network analysis of the ECG (accuracy 86%) matched expert human electrophysiologists. Incorporation of artificial intelligence enhanced ECG (AI-ECG) analysis could help guide treatment decisions and procedural planning in patients with organized atrial tachyarrhythmias.

### Neural networks for arrhythmia classification

Previous studies have trained neural networks with very high accuracy for multiclass arrhythmia detection against human ECG interpretation as the gold standard. Hannun *et al.*^12^ achieved an AUC of 0.97, Zhu *et al.*^13^ an AUC of 0.98, and Ribeiro *et al.*^11^ F1 scores of over 80%. Neural networks in this context will therefore at best match expert human performance, which may be fallible. Our study used the findings of the invasive EP study as the gold standard, allowing the neural network to ‘learn’ from the most accurate means of arrhythmia diagnosis. Using this approach, our neural network was able to match expert electrophysiologist performance, despite a relatively small dataset of patients. The utility of the previously reported models^11–13^ are scenarios where expert human interpretation of the ECG is not available, for example in rural or remote areas where telemedicine is important. In contrast, our approach provides utility in centres where ablation procedures are carried out, to support the electrophysiologist with AI-ECG analysis.

### Previous studies investigating AT mechanism from the ECG

Luongo *et al.*^25^ recently described a hybrid in silico and clinical ECG method to train a decision tree classifier to distinguish between CTI-dependent atrial flutter, mitral isthmus dependent flutter, and other left atrial flutters. In order to evaluate the performance of their model for the binary classification task of CTI-dependent flutter vs. non-CTI-dependent, the mitral isthmus dependent flutter and other left atrial flutter groups can be combined. For the binary task, their model had 87% accuracy on a per patient basis, and this is overall very similar to our study. However, there are several important differences between their study and the current work. Their study included only CTI-dependent flutter or macro-reentrant left atrial flutters, while the current study included a wide range of ATs, both left and right atrial, focal, and re-entrant. Another key difference is that their decision tree classifier requires manual segmentation of the ECG to reduce the presence of QRS-T complexes and to select a segment for analysis that starts and ends at the same point of the F-wave. A manual approach can be both time consuming and also may introduce human error or inter-operator variability into the model performance. By contrast, the CNN approach used in the current work is entirely automated, does not require human input and takes a complete digitized 12-lead ECG as input.

Other previous studies have described manual methods to classify AT mechanisms from the ECG. Pascale *et al.*^26^ described various ECG features with high specificity for different AT mechanisms, however with overall low sensitivities (25–59%). Mohammadi *et al.*^27^described the use of independent component analysis to classify focal AT to left or right atrial source in 32 patients with a mean accuracy of 93%. Manual algorithms for focal ATs have also been described.^28^ Neural networks have not to our knowledge been used to investigate AT mechanism. Manual algorithms in particular can be challenging and time consuming, with inter operator variability. Our neural network, on the other hand, is able to give a rapid and accurate diagnosis with no manual processing steps.

### Potential clinical role for AT/AFL classifier

An accurate AI-ECG tool for classification of CTI-dependent AFL vs. other ATs has the potential to be a key tool in guiding treatment decisions. Although CTI-dependent AFL often has a classical ECG appearance, our survey of expert electrophysiologists demonstrates that the distinction is not always clear. Given the high success rates of ablation for CTI-dependent AFL,^8^ patients and physicians are likely to have a lower threshold for CTI ablation than the ablation of other ATs. Additionally, accurate knowledge of tachycardia mechanism can help plan procedures, including the provision of general anaesthesia and TOE or intracardiac echocardiography.^26^ In cases where the atrial arrhythmia is paroxysmal, non-CTI-dependent ATs require induction of the tachycardia to map the mechanism.^29^ CTI-dependent AFL however can be treated with empirical ablation,^30^ and AI-ECG tool could therefore be used to facilitate empirical CTI ablation where the tachycardia is not inducible during the procedure. Patient counselling could also be more specific regarding success rates and complication rates. Lastly, accurate upfront knowledge of tachycardia mechanism has the potential to shorten procedure time.

Potential workflows include implementation of AI-ECG in an outpatient clinic consultation. This could be used by non-electrophysiologists, if CTI-dependent atrial flutter is identified, referral on to electrophysiologists for an ablation could be favoured. The electrophysiologist could also utilize the algorithm to favour the invasive approach where atrial flutter is diagnosed, or for more detailed procedure planning or patient consent. In our test set there were no cases where both the model and electrophysiologist had the incorrect diagnosis. Additionally, the prediction accuracy where electrophysiologists and the model agreed was 100%. The model could therefore be used to complement electrophysiologist interpretation.

### Saliency mapping to improve acceptance of artificial intelligence into clinical care

Humans are, with good reason, hesitant to adopt artificial intelligence (AI) as a ‘black box’ into clinical care. There are several examples in the literature of AI ‘shortcuts’ that can lead to improper features being used for diagnoses. Winkler *et al* described how skin markings in melanoma images can lead a CNN to associate skin markings with pathology.^31^ Explainable AI is key to adoption of AI into clinical practice,^32^ and to this end, saliency mapping is a useful way to ensure that unexpected features are not being used for classification. The saliency maps in our study show that the CNN is using the expected parts of the ECG, the atrial activity, and not unexpected segments such as the QRS, to classify the ECGs. This provides greater confidence that the model could be applied clinically.

## Limitations

Although our study demonstrates equivalence to electrophysiologist performance, the model presented is a proof of concept, derived from a relatively small training dataset, due to the need for an invasive EP study in all cases. Given the very large datasets often used for deep learning applications, it is likely a larger dataset would further increase the model accuracy to surpass humans. Our data were from a single centre with a single EP recording system, and external validity requires confirmation with analysis of ECGs from other centres and recording systems. The confirmation of CTI-dependence was the primary goal in the EP studies that were undertaken for suspected CTI-dependent AFL. Of those with CTI-dependent AFL, only a small minority of patients had 3D mapping or a multipolar catheter placed in the right atrium. Therefore, for the majority of cases, clockwise vs. counter-clockwise CTI-dependent AFL was not distinguished. However, as the treatment for both is the same (CTI line ablation), we do not feel this limitation affects the clinical utility of our model. In clinical practice, humans would use other clinical information to influence the pre-test probability, e.g. prior left atrial ablations or atriotomy increases the chance of non-CTI dependence. It is therefore possible that human performance could surpass the model if provided with this information, however, equally, the neural network could be trained to utilize this information. A limitation of the saliency mapping method used in our analysis is that we are unable to assess the relative importance of each ECG lead to the final model classification, and we therefore are unable to determine which ECG leads were most important in assessing each classification decision. A limitation of deep learning in general is the inability to fully understand the factors used to make a decision. In this case we are unable to identify the exact morphological characteristics used by the model, and therefore cannot compare the ECG features used in our model to that of Luongo *et al.*^25^ or Pascale *et al*.^26^ To analyse an ECG with our neural network, a digitized ECG is required, this is not currently available outside of an EP lab in many centres. This is an important limiting factor; one potential solution would be the incorporation of a scanned ECG to digital signal converter. The input to our neural network must be an ECG of an organized atrial arrhythmia. We did not collect data from other rhythms such as atrial fibrillation, sinus tachycardia, or sinus rhythm, and therefore, ECG inputs require human involvement to ensure they are appropriate. For example, coarse fibrillatory waves in AF may appear to be organized. A potential solution would be to implement our proposed model after other existing models have excluded other arrhythmia diagnoses such as AF. We did not have sufficient data of each subclass of AT (e.g. right vs. left) to train a neural network for this task.

## Conclusion

We describe the first CNN trained to differentiate CTI-dependent AFL from other ATs, without the need for any user input. We found that our model matched expert electrophysiologist performance. Automated AI-ECG analysis could help guide treatment decisions and plan ablation procedures for patients with organized atrial arrhythmias.

## Supplementary material

Supplementary material is available at *European Heart Journal – Digital Health* online.

## Supplementary Material

ztac042_Supplementary_Data

## Data Availability

The datasets generated or analysed or both during this study are not publicly available owing to ethical restrictions.
