# Supplementary material for: Artificial intelligence-enabled electrocardiogram to distinguish cavotricuspid isthmus dependence from other atrial tachycardia mechanisms
Source: Eur Heart J Digit Health. 2022 Aug 17;3(3):405–14. doi: 10.1093/ehjdh/ztac042 (PMC9708023; doi:10.1093/ehjdh/ztac042)
Supplement: ztac042_Supplementary_Data [file ztac042_supplementary_data.pdf]

Artificial intelligence-enabled electrocardiogram to distinguish cavotricuspid isthmus dependence from other atrial tachycardia mechanisms

Supplementary material

Supplementary methods

ECG pre-processing

A 5s segment duration was selected as these performed better than 10s recordings on preliminary analyses. Subsampling the ECG recording is an established methodology to reduce the number of model parameters ((1)) and can be seen as a form of data augmentation to allow the network to learn from the subtle variations in each sub sample. Given the current classification task was to diagnose arrhythmia mechanisms, ECG durations shorter than 5s were considered to be too short and therefore were not examined. A detailed comparison of ideal subsampling durations is beyond the scope of this manuscript.

Digital 12-lead ECGs were recorded using the EP recording system (LABSYSTEM™ PRO (Boston Scientific)) as previously described (2). ECGs were downsampled to 400hz as this was the lowest input sampling rate of the candidate architectures that used a 12 lead ECG input (Hannun et al (3) used a single lead ECG input sampled at 200hz). Comparison of 400hz sampling rate vs. 500hz on validation data showed no differences in model accuracy, therefore 400hz was chosen to reduce the data input size and number of model parameters, which consequently would reduce training time Digital ECG recordings from outside the EP lab were not available, therefore we were unable to compare performance of the network with ECGs not recording the EP recording system, however other than the higher sampling rate there is no major differences in how the ECG is recorded.

Although a 12 ECG leads ECG uses data from only 8 independent leads, all 12 leads were used as input in the neural network to match to candidate network architectures.

Architecture selection and hyperparameter optimization

1-dimensional convolutional neural network (CNN) architectures were implemented. The ECG signal is treated as a 1-dimensional time series with 12 channels. In order to fairly compare each candidate architecture, Keras tuner was used to perform a grid search to identify optimal hyperparameters for each architecture. Candidate architectures were: Zhu et al(4), Hannun et al(3), Attia et al (1), Ribeiro et al (5) Resnet 18, 34 and 50(6). Resnet 34 and Resnet 50 architectures did not generalise well during hyperparameter tuning, likely due to their large size, and therefore were excluded from further testing. Architectures were then compared to identify optimal performance as shown in supplementary table S1. Where architectures had similar performance, small networks with fewer parameters were preferred.

The final architecture was a modified version of that used by Attia et al (1). Their neural network architecture was used for a 2 second ECG segment, sampled at 500hz and zero padded to a final length of 1024. In contrast, given that our network is designed to make a rhythm diagnosis, we elected to use a longer ECG segment. Our network therefore takes an input of a 5 second, sampled at 400hz and zero padded to a final length of 2048. During hyperparameter tuning, setting dropout to 0 resulted in the highest validation set accuracy. Therefore the dropout layer after the fully connected layer was removed, which was another difference to the network

architecture of Attia et al. Lastly, the kernel size and number of filters for the last convolutional layer was not specified. A kernel size of 3 with 128 filters were chosen for the final convolutional layer in line with common convention of increasing the number of filters deeper into the network. Importantly, the final architecture was decided purely based on performance on the validation set.

Explainable artificial intelligence predictions

Saliency mapping has been used to explain CNN models ((2, 7, 8)). Grad-CAM is another method that has been used for this purpose (9). Grad-CAM identifies the last convolutional layer and investigates the gradients flowing into the layer. This works well for providing coarse localisation for images where a common kernel size is 5x5. This method however was not applicable to our model architecture given the small kernel size of 3 in the last convolutional layer. This kernel size results in the Grad-CAM mapping being far too coarse to be of any use. Other related methods such as guided Grad-CAM have been shown to be unreliable (10).

Supplementary table S1

Candidate neural network architectures

	Parameters	Accuracy (%)
Attia	120,737	85.4
Ribeiro	1,733,613	84.9
Hannun	2,942,177	78.8
Resnet18	4,350,465	78.4
Zhu	4,736,129	78.2

Supplementary table S2

Patient arrhythmia and clinical characteristics in training and test sets
CTI: cavotricuspid isthmus

Training set	AFL	AT
N	118	113
Right atrial origin, n (%)	N/A	26 (23%)
Left atrial origin, n (%)	N/A	87 (77%)
Previous left atrial ablation, n (%)	2 (2%)	64 (57%)
Previous CTI ablation, n (%)	5 (4%)	21 (19%)
Previous cardiac surgery, n (%)	3 (3%)	14(12%)
Test set		
N	29	28
Right atrial origin, n (%)	N/A	8 (28%)
Left atrial origin, n (%)	N/A	20 (71%)
Previous AF ablation, n (%)	0 (0%)	13 (46%)
Previous CTI line ablation, n (%)	2 (7%)	8 (29%)
Previous cardiac surgery, n (%)	0 (0%)	5 (18%)

Supplementary table S3

Contingency table of electrophysiologist (EP) vs model performance

	EP consensus correct	EP consensus incorrect
Model correct	38 (67%)	11 (19%)
Model incorrect	8 (14%)	0 (0%)

Supplementary figure S1

Flow chart of steps from patient screening to prepared ECG inputs. CTI: cavotricuspid isthmus, AT: non-CTI dependent atrial tachycardia, AFL: CTI-dependent atrial flutter

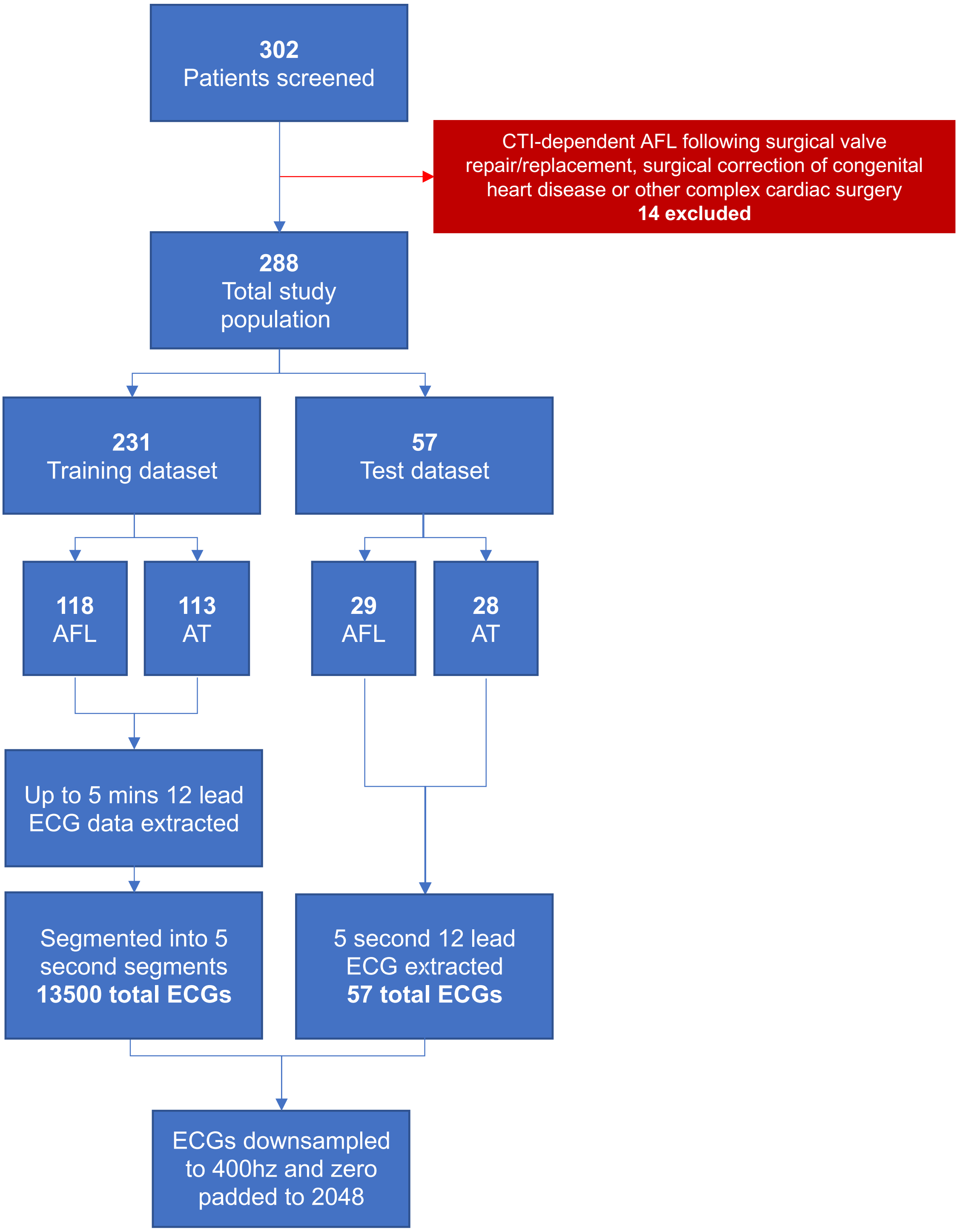

Supplementary figure S2

Flow chart of data flow from derivation of training dataset to model training. Testing dataset remains separate throughout and is used only for model evaluation

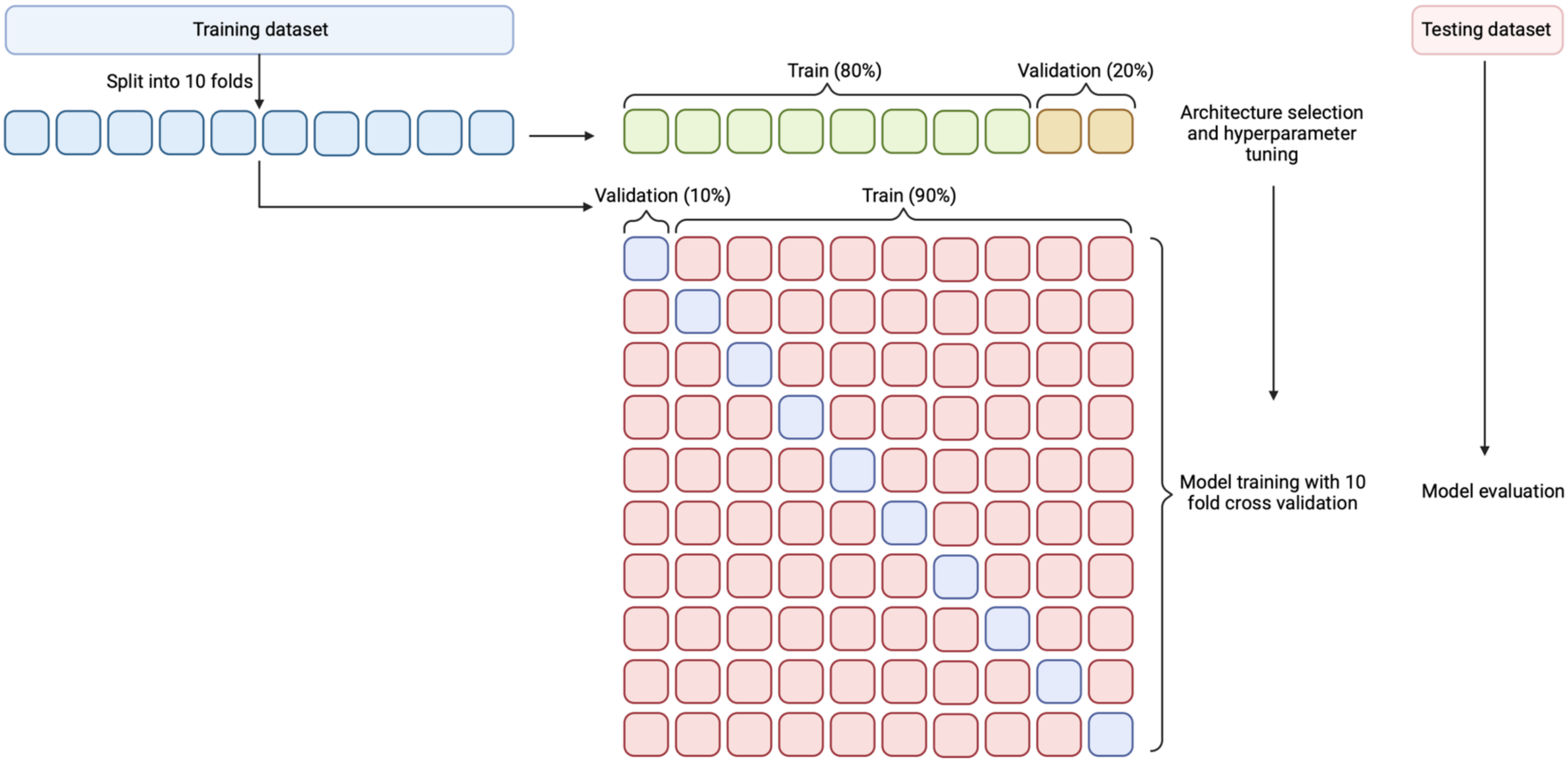

Supplementary figure S3

Example survey question using an online survey platform. Respondents were provided with a 12 lead ECG and asked to make a binary choice and diagnose with CTI dependent flutter or Non-CTI dependent atrial tachycardia

11. Based on the ECG, what is the most likely diagnosis?

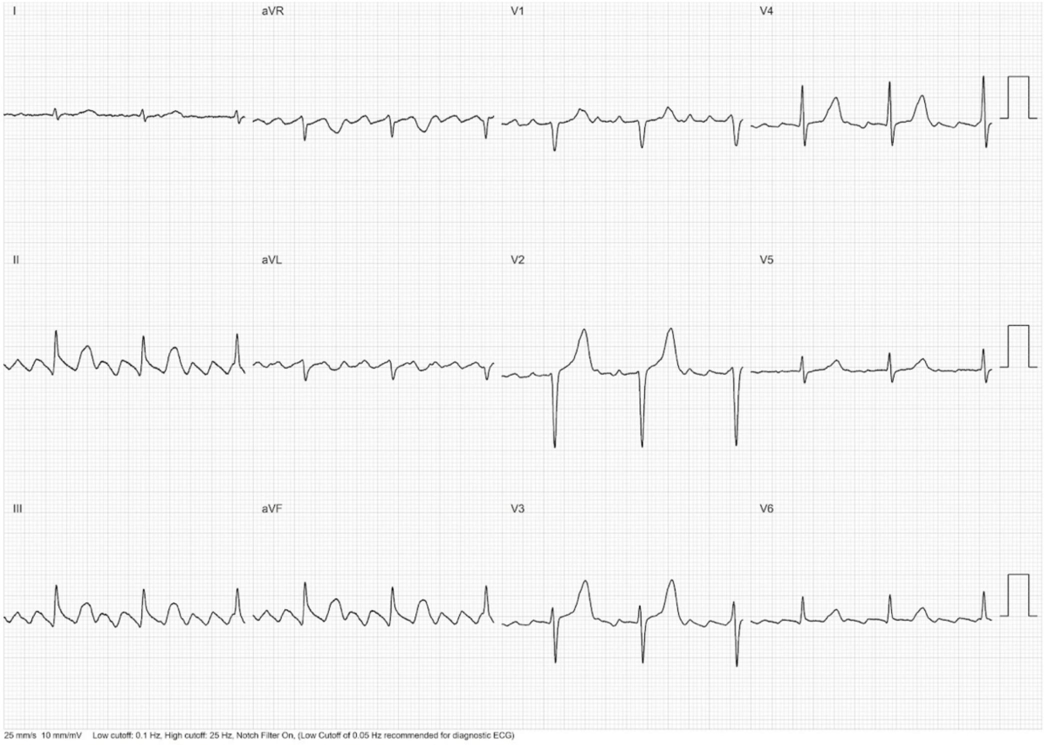

- ○ CTI dependent flutter
- ○ Non-CTI dependent atrial tachycardia

Supplementary figure S4

Example saliency maps where model incorrectly classified as non-CTI dependent atrial tachycardia, correct class was CTI dependent atrial flutter

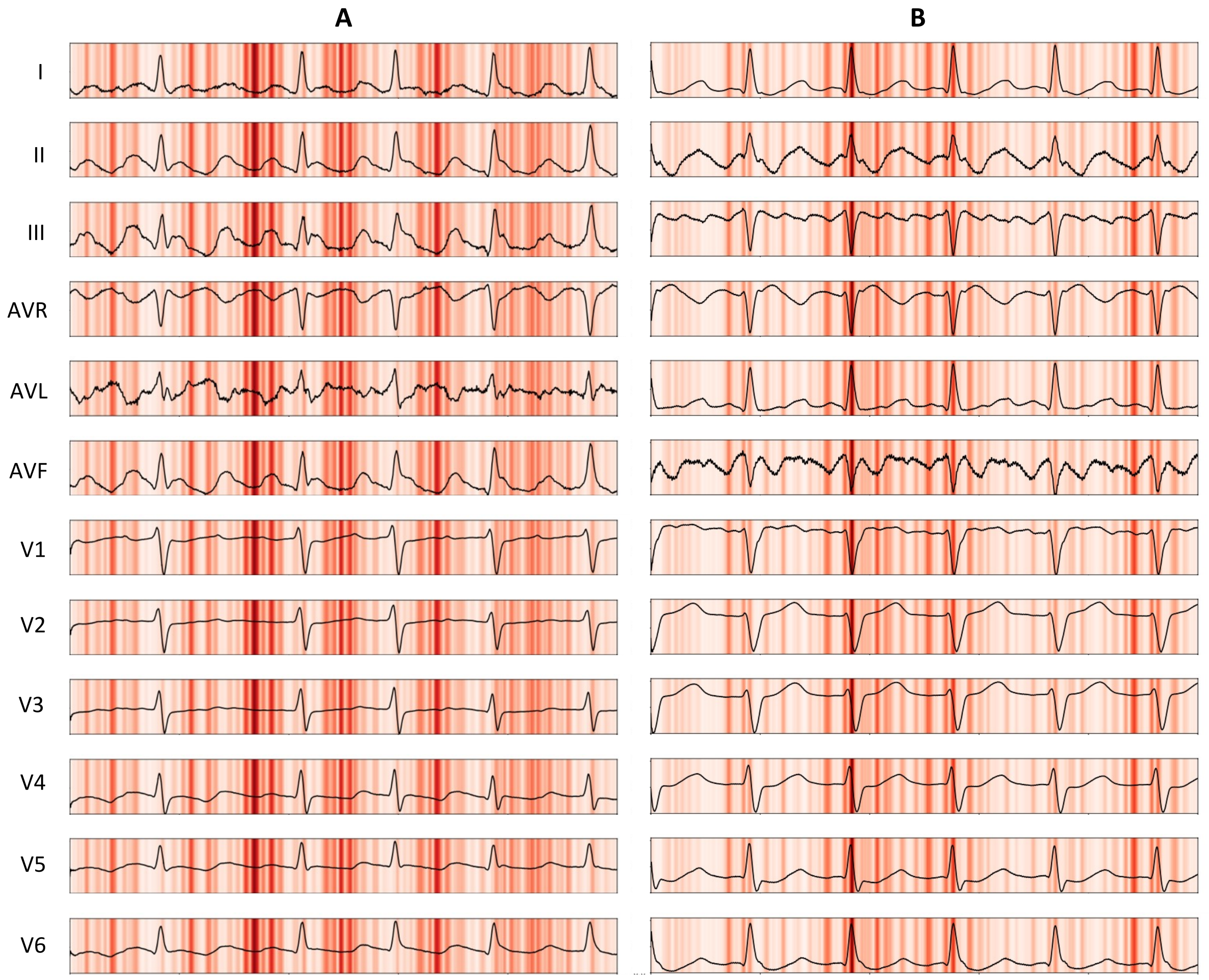

Supplementary figure S5

Example saliency maps where model incorrectly classified as CTI dependent atrial flutter, correct class was non-CTI dependent atrial tachycardia

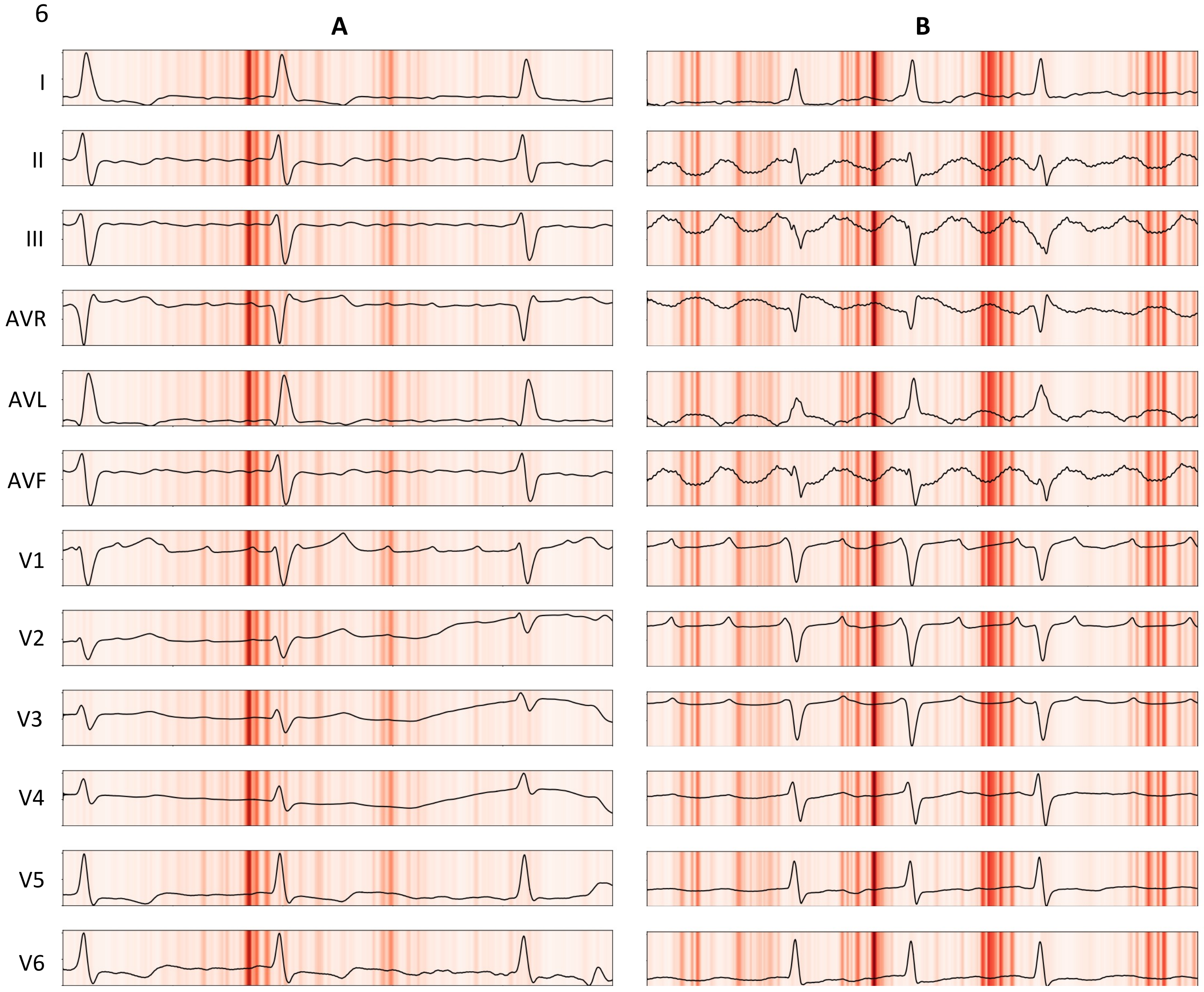

Supplementary figure S6
Confusion matrix demonstrating human consensus performance at classifying between the two groups. AFL and AT cases were correctly classified at 96.4% and 65.5%, respectively
AT: non-CTI dependent atrial tachycardia, AFL: CTI-dependent atrial flutter

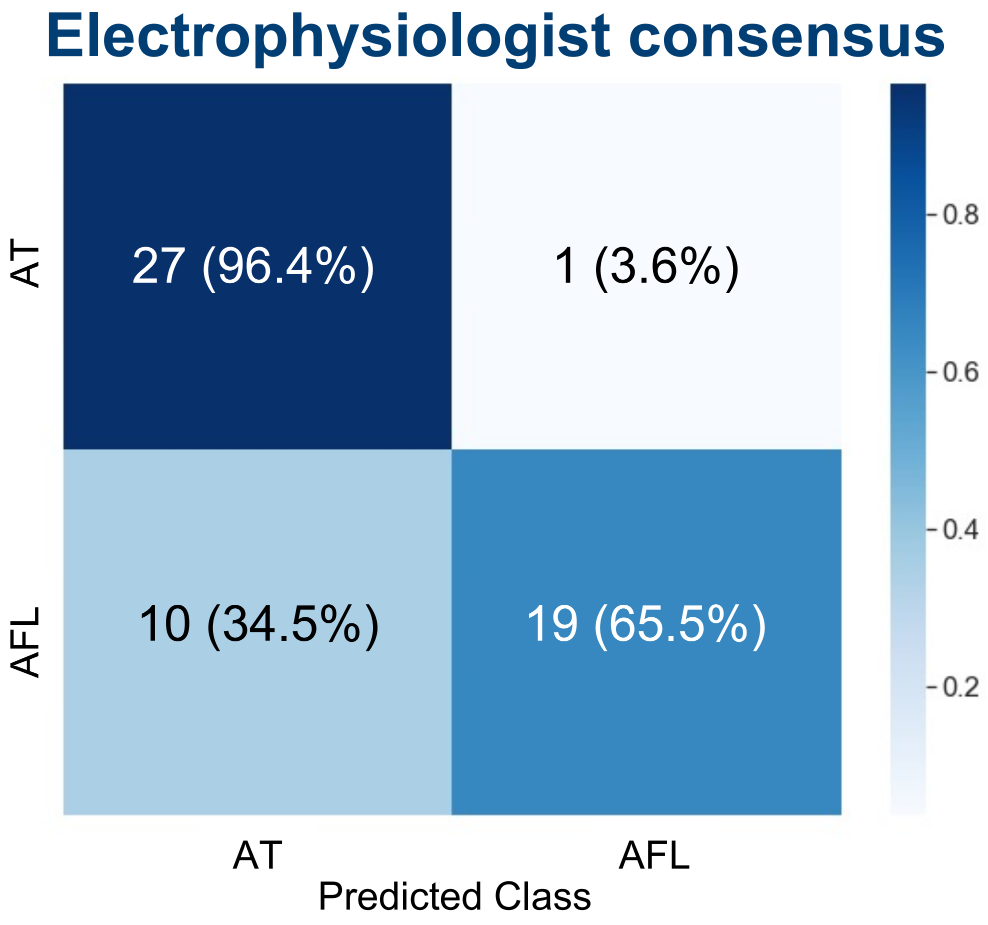

Supplementary figure S7

Examples of cases where both the model and the majority of human experts were correct

A) CTI-dependent flutter – Diagnosis confirmed with 3D mapping and termination during CTI line ablation

B) Non-CTI dependent atrial tachycardia – Previous mitral valve replacement, tricuspid valve annuloplasty and CTI line ablation. 3D mapping used to diagnose re-entrant circuit at site of previous atriotomy, tachycardia terminated during ablation

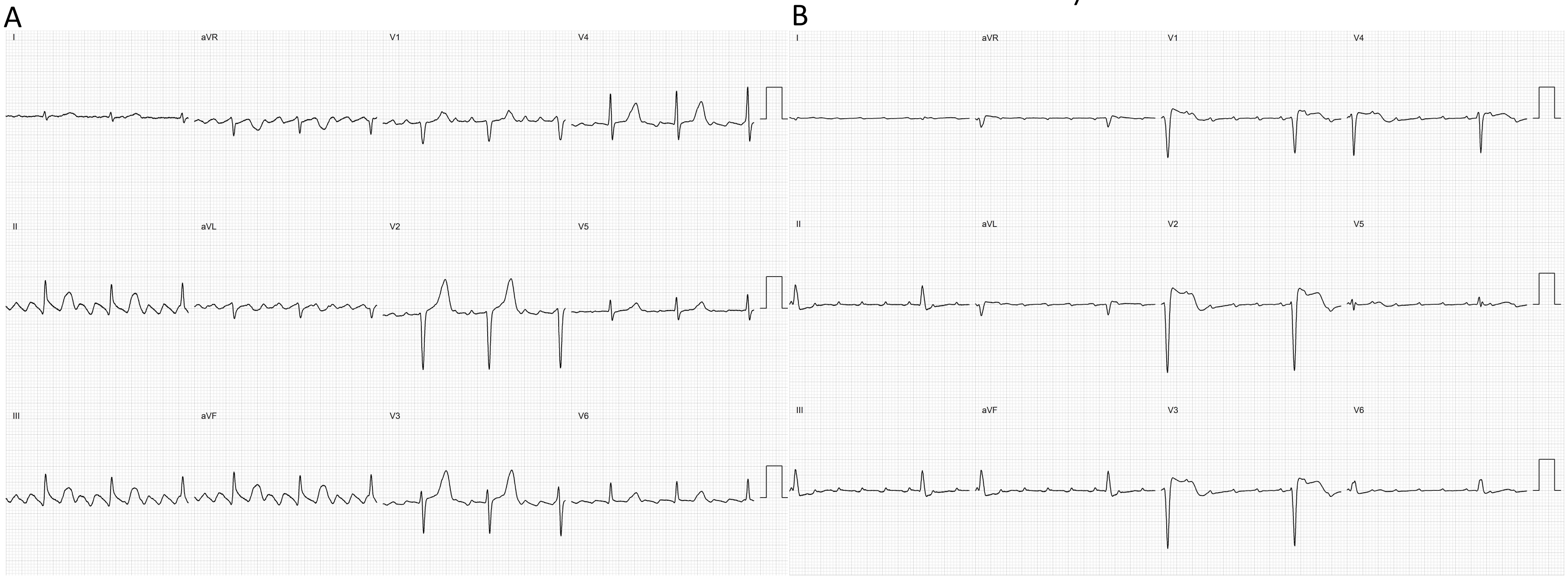

Supplementary figure S8

Examples of cases where the model was correct and majority of human experts were incorrect

A) CTI-dependent atrial flutter - confirmed by entrainment

B) Non-CTI dependent atrial tachycardia - CTI line initially performed with no change in tachycardia, subsequent 3D mapping demonstrated a focal tachycardia originating from the coronary sinus ostium, tachycardia terminated with ablation at this site

A B

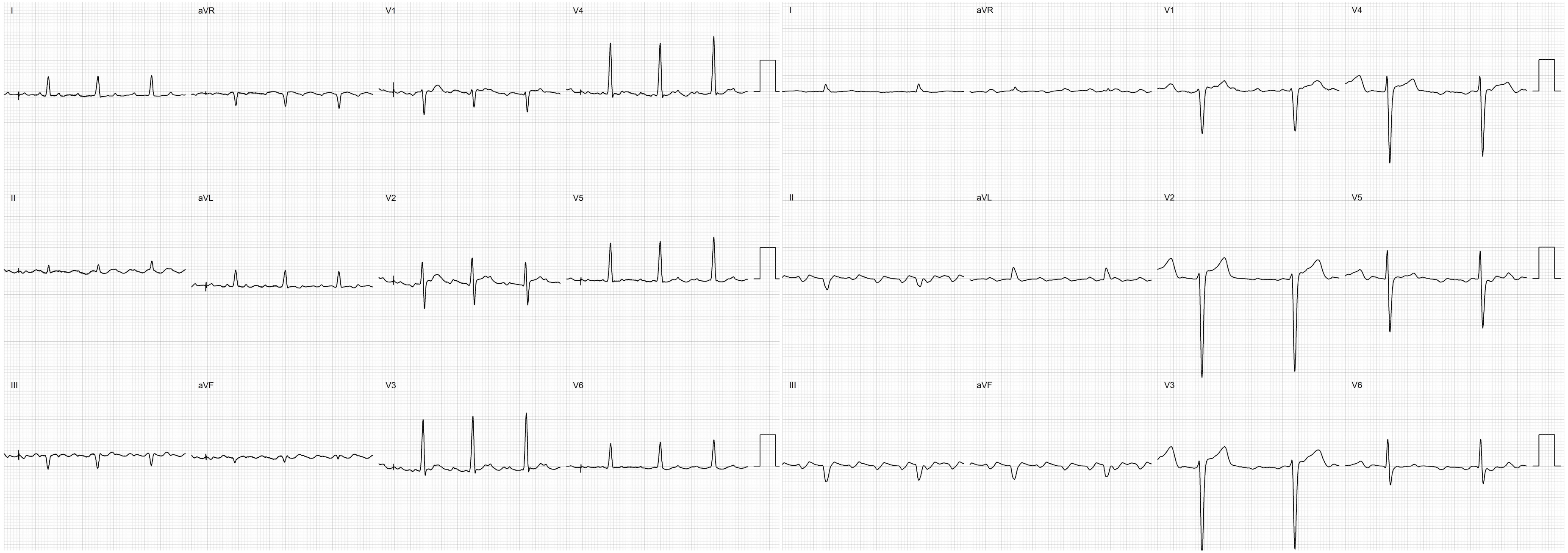

Supplementary figure S9

Examples of cases where the model was incorrect and majority of human experts were correct

A) CTI-dependent flutter – activation consistent with clockwise CTI dependent AFL based on Halo catheter activation. Diagnosis confirmed with termination of tachycardia during CTI line ablation

B) Non-CTI dependent AT – Previous CTI line, 3D mapping used to diagnose localised reentry on left atrial side of atrial septum, tachycardia terminated during ablation

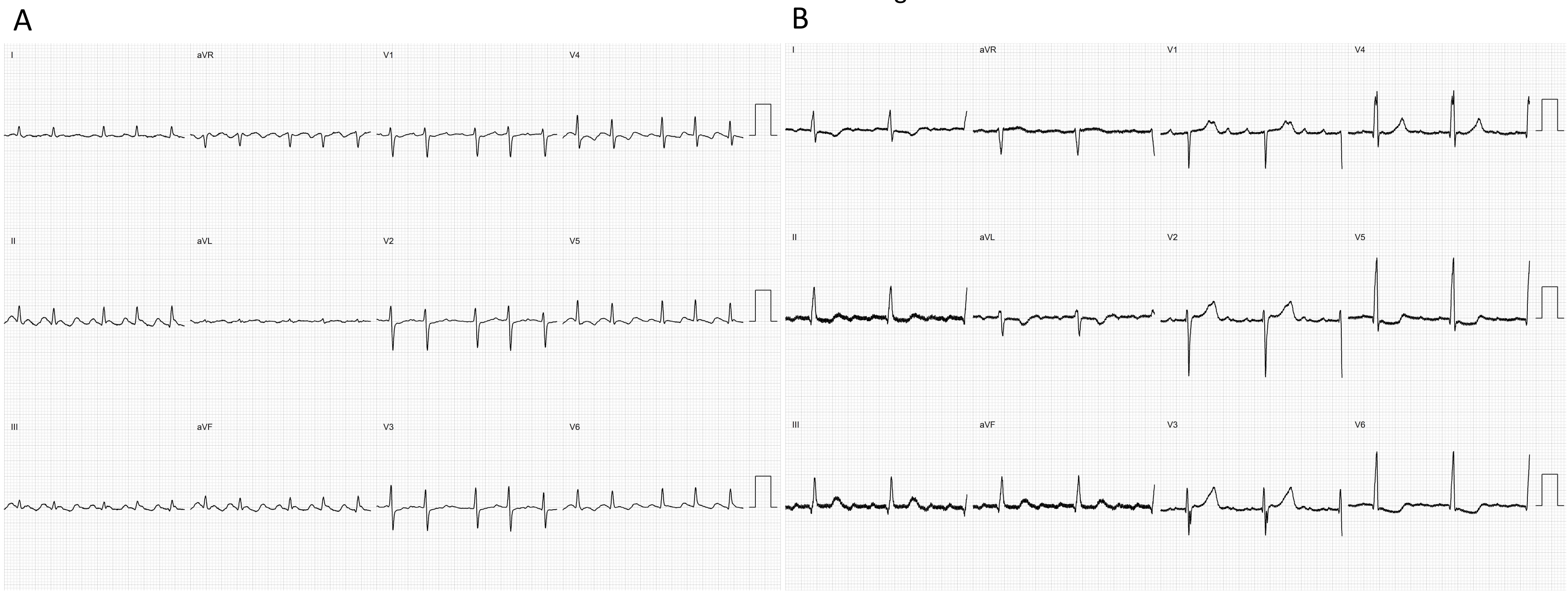

References

1. Attia ZI, Kapa S, Lopez-Jimenez F, McKie PM, Ladewig DJ, Satam G, et al. Screening for cardiac contractile dysfunction using an artificial intelligence-enabled electrocardiogram. Nat Med. 2019;25(1):70-4.
2. Arnold AD, Howard JP, Gopi A, Chan CP, Ali N, Keene D, et al. Discriminating electrocardiographic responses to His-bundle pacing using machine learning. Cardiovascular Digital Health Journal. 2020;1(1):11-20.
3. Hannun AY, Rajpurkar P, Haghpanahi M, Tison GH, Bourn C, Turakhia MP, et al. Cardiologist-level arrhythmia detection and classification in ambulatory electrocardiograms using a deep neural network. Nat Med. 2019;25(1):65-9.
4. Zhu H, Cheng C, Yin H, Li X, Zuo P, Ding J, et al. Automatic multilabel electrocardiogram diagnosis of heart rhythm or conduction abnormalities with deep learning: a cohort study. The Lancet Digital Health. 2020;2(7):e348-e57.
5. Ribeiro AH, Ribeiro MH, Paixao GMM, Oliveira DM, Gomes PR, Canazart JA, et al. Automatic diagnosis of the 12-lead ECG using a deep neural network. Nat Commun. 2020;11(1):1760.
6. He K, Zhang X, Ren S, Sun J, editors. Deep Residual Learning for Image Recognition. 2016 IEEE Conference on Computer Vision and Pattern Recognition (CVPR); 2016 27-30 June 2016.
7. Cohen-Shelly M, Attia ZI, Friedman PA, Ito S, Essayagh BA, Ko WY, et al. Electrocardiogram screening for aortic valve stenosis using artificial intelligence. Eur Heart J. 2021;42(30):2885-96.
8. Lima EM, Ribeiro AH, Paixao GMM, Ribeiro MH, Pinto-Filho MM, Gomes PR, et al. Deep neural network-estimated electrocardiographic age as a mortality predictor. Nat Commun. 2021;12(1):5117.
9. Selvaraju RR, Cogswell M, Das A, Vedantam R, Parikh D, Batra D. Grad-CAM: Visual Explanations from Deep Networks via Gradient-Based Localization. International Journal of Computer Vision. 2019;128(2):336-59.
10. Adebayo J, Gilmer J, Muelly M, Goodfellow I, Hardt M, Kim B. Sanity checks for saliency maps. Advances in neural information processing systems. 2018;31.
